# Positive physician perceptions of structured lung ultrasound (LUS) training in interstitial lung disease (ILD): a post-workshop survey study

**DOI:** 10.1007/s00296-026-06191-4

**Published:** 2026-06-03

**Authors:** Mirja M. Wirtz, Stefanie Derenthal, Andrea Studnicka-Benke, Michael Studnicka

**Affiliations:** Department of Pneumology, Salzburger Landeskliniken, Paracelsus Medical University, Salzburg, Austria

**Keywords:** Interstitial lung disease, Surveys and questionnaires, Point-of-care testing, Ultrasonography, Continuing medical education, Program evaluation

## Abstract

Current guidelines recommend structured training in lung ultrasound (LUS) for interstitial lung disease (ILD). Therefore, this exploratory study evaluated physician perceptions, perceived educational value, and self-reported applicability of a structured LUS workshop involving patients with ILD. A six-hour LUS workshop combining didactic lectures with supervised hands-on training on ILD patients was conducted for a group of actively practicing rheumatologists and pulmonologists. Workshop evaluation was performed using an anonymous questionnaire capturing participants’ demographics and pre-course LUS practice, course satisfaction, and perceived barriers to using LUS routinely in ILD care. Ten physicians, predominantly experienced rheumatologists with regular exposure to ILD patients, attended the workshop. Pre-course LUS was infrequent, with only one participant reporting regular use. Following the workshop, all participants reported perceived improvement in LUS knowledge and increased self-confidence in recognizing typical sonographic features of ILD. The workshop was universally rated as clinically relevant, and nine of ten attendees expressed intention to integrate LUS into their clinical practice. Lack of experience and time constraints were identified as main perceived barriers, whereas standardized protocols and focused training were viewed as important facilitators for clinical adoption of LUS in ILD. This exploratory study suggests that a concise, structured LUS workshop involving patients with ILD was perceived as clinically relevant and educationally valuable by participating physicians.

**Supplementary Information:**

The online version contains supplementary material available at 10.1007/s00296-026-06191-4.

## Introduction

Interstitial lung diseases (ILDs) comprise a heterogeneous group of parenchymal lung disorders [1]. A substantial proportion of ILD patients follow a fibrosing course which is associated with progressive functional decline, high morbidity, and increased mortality [2, 3]. Early identification of ILD is therefore crucial, as timely therapeutic interventions, including anti-inflammatory and antifibrotic treatment, can slow disease progression and improve outcomes [4, 5]. 

High-resolution computed tomography (HRCT), followed by a multidisciplinary discussion, remains the reference standard for diagnosing and assessing ILDs [6]. However, repeated HRCT examinations are associated with considerable costs and cumulative radiation exposure, highlighting the need for complementary, non-invasive and readily available tools suitable for screening and longitudinal monitoring [7]. 

There is growing interest in lung ultrasound (LUS) as such a point-of-care tool for ILDs, given its availability, low cost, non-invasive nature, and lack of radiation compared with repeated HRCT [5–7]. The LUS hallmark of ILD is considered an increased number of vertical artefacts (also known as B-lines or “comet tails”) along with pleural irregularity [8]. Despite emerging data suggesting potential utility of LUS in ILD, existing recommendations do not support its routine clinical use yet due to sparse and heterogeneous data generating only low certainty of evidence [5, 6, 8]. 

Accordingly, and because limited experience, insufficient training, and time constraints are frequently cited barriers to clinical use of LUS [9], the current ERS/EULAR guidelines for connective tissue disease-associated ILD (CTD-ILD) recommend the development of standardized LUS protocols and the organization of structured training programs for health care providers [5]. While ultrasound workshops have been associated with educational benefit in other clinical contexts [9–11], data on concise, structured LUS training specifically focusing on ILD, particularly regarding physician appraisal and perceived clinical value, are scarce.

In line with these recommendations and to support future educational strategies and broader clinical adoption of LUS, the present study is the first to our knowledge to explore physician perceptions, perceived educational value, and self-reported applicability of a targeted LUS workshop involving actual ILD patients in a clinically active physician cohort.

## Methods

### Study design

Our study was designed as an exploratory, questionnaire-based post-workshop survey study evaluating physician perceptions following a structured LUS workshop.

### Participants

Local and regional rheumatologists and pulmonologists from both hospital-based and outpatient settings were invited to attend through distribution channels of the organizing academic faculty and industry. Participation was purposefully limited to ten attendees to ensure intensive hands-on training and allocated on a first-come, first-served basis.

### Workshop structure

The workshop was conducted on January 30th, 2026, as a one-day, six-hour in-person training program that combined concise didactic sessions with intensive hands-on training. Sessions on LUS were facilitated by two board-certified internists with subspecialty training in rheumatology and cardiology, both having extensive expertise in LUS. The course consisted of a 30-minute didactic session covering basic ultrasound physics, key LUS techniques (including probe positioning, artifact and anatomical landmark recognition as well as clinical-pathological correlation), and characteristic LUS findings in ILD (presence of B-lines and pleural line abnormalities). Participants then completed two supervised 90-minute hands-on sessions in equally sized groups on consenting patients with known ILD (one representing a nonspecific interstitial pneumonia (NSIP) and one a usual interstitial pneumonia (UIP) pattern). Before concluding with a 30-minute interactive and case-based session highlighting common pulmonary and cardiological conditions in which LUS aids diagnosis, participants attended a structured 30-minute lecture on ILD. This lecture, delivered by an experienced, board-certified pulmonologist, provided a comprehensive overview of the disease, including epidemiology, the importance of early detection, treatment considerations, prognosis, and current screening recommendations. An industry-supported framework served as the starting point for development of the workshop. However, all educational content was independently developed by the academic faculty without involvement of the industry.

### Workshop evaluation

The workshop was evaluated using a questionnaire developed by the academic faculty (see English-language questionnaire provided in Supplement 1). The questionnaire was designed based on previously published ultrasound education surveys [11, 12] and adapted to the specific context of LUS in ILD. Prior to workshop implementation, the draft questionnaire underwent internal review and pilot pretesting for face validity, clarity, content relevance, and consistency with the aims of the study.

Participants completed the anonymous 18-item survey immediately after the workshop, either electronically via an online platform (Microsoft Forms) or on paper, according to their preference. The questionnaire captured key demographic and professional characteristics, prior ultrasound and LUS practice, perceived educational value of the workshop, self-reported confidence in recognizing typical sonographic ILD findings, future applicability of LUS, and perceived barriers and facilitators to its adoption in clinical practice. It comprised nine categorical single-choice items, seven Likert-scale items (ranging from full agreement to full disagreement), one multiple-selection item, and one open-ended question.

### Statistics

All data were analyzed descriptively, in line with the exploratory design of the study. Due to the small sample size, quantitative and Likert-scale responses are presented as frequencies only. Free-text responses were thematically grouped and quantified. Graphs were created using Microsoft Office.

## Results

### Participant profile

Ten physicians attended the LUS workshop. All completed the post-workshop survey. There were eight female and two male participants, of which seven were aged ≥ 45 years. Most workshop attendees (eight in total) were internists with a specialization in rheumatology, whereas the remaining two participants were specialized in pulmonology. All attendees were based in a hospital setting, with two participants also practicing in an outpatient setting. Eight of ten participants were highly experienced, having worked in their profession for at least ten years. Notably, four attendees reported more than 20 years of experience. Nine of ten participants reported providing care for patients with diagnosed ILD or at risk of its development at least occasionally (six physicians reported regular and three physicians occasional ILD patient care). Demographic and professional characteristics of participants are summarized in Table 1.

**Table 1:** Demographic and professional characteristics of workshop participants

	Participants (*n* = 10)
*Gender*	
Female	8
Male	2
*Age group*	
< 30 years	1
30 to 44 years	2
45 to 59 years	6
> 60 years	1
*Medical specialty*	
Internal medicine and rheumatology	8
Pulmonology	2
*Work environment*	
Hospital-based	8
Outpatient-based	0
Both	2
*Years of clinical experience*	
< 5 years	1
5 to 9 years	1
10 to 14 years	2
15 to 19 years	2
> 20 years	4
*Frequency of ILD patient care*	
Regularly	6
Occasionally	3
Rarely	0
Never	1

### Ultrasound practice among participants prior to the workshop

**Fig. 1:**
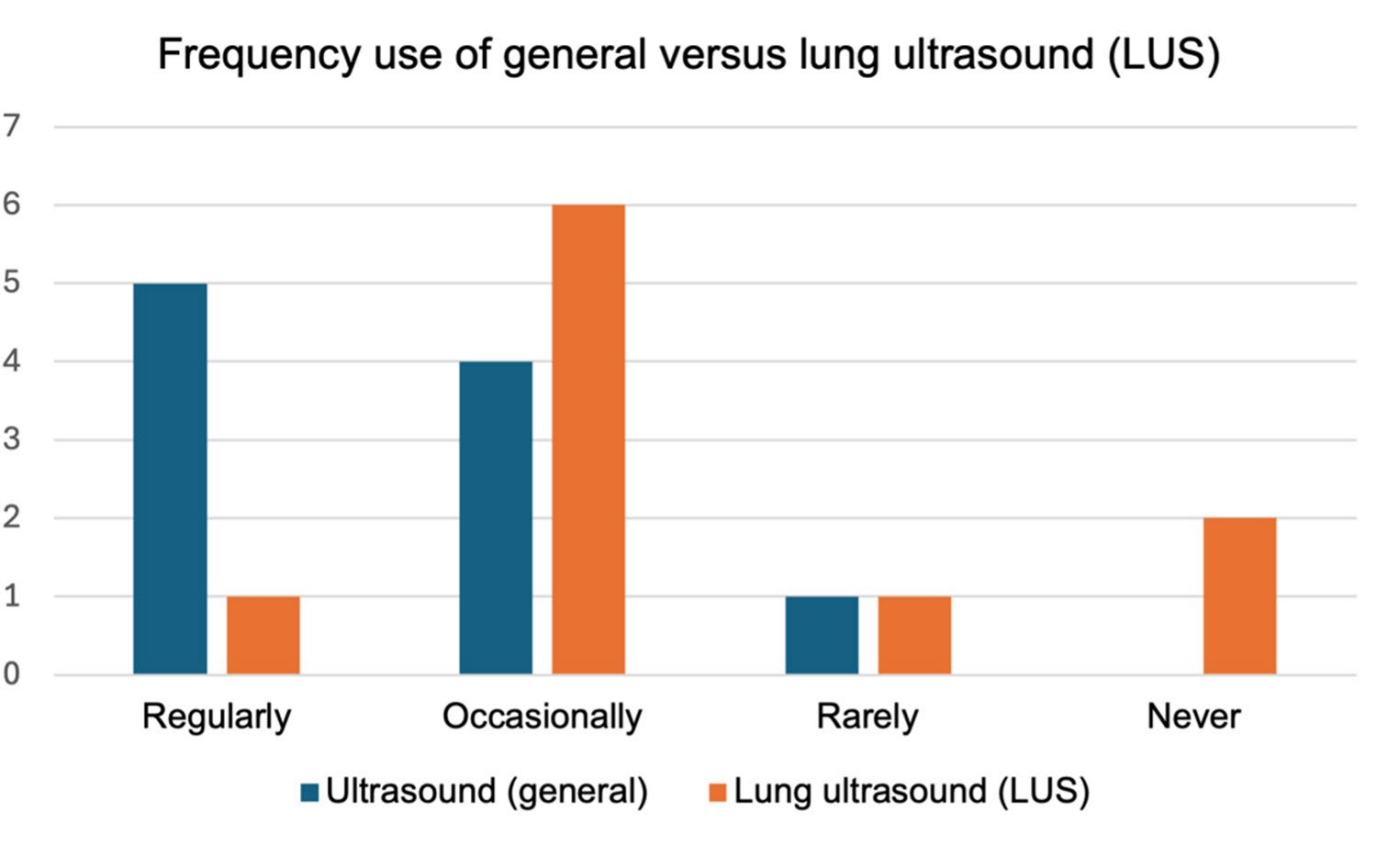
Participants’ frequency use of general versus lung ultrasound (LUS)

Ultrasound in general was indicated to be used in clinical practice at least occasionally by nine participants (five reported regular use, four occasional use, and only one physician reported rare use). In contrast, LUS was used at least occasionally by only seven participants, with just one attendee reporting regular use (Fig. 1). There was variability in the amount of time participants felt they could allocate in their current practice to an additional examination such as LUS: While two indicated less than 5 min and another two more than 15 min, most attendees (five) reported being able to dedicate 5–9 min for such an examination.

### Participant reaction to and satisfaction with the workshop

All ten participants rated the content of the workshop as useful and easy to understand. Furthermore, all participants considered the course to be relevant to their clinical practice, with nine physicians fully and one physician rather agreeing. All workshop attendees reported a significant improvement in their LUS knowledge through the workshop (eight fully agreed, two rather agreed). In addition, all participants expressed their intention to integrate LUS into their clinical practice (nine fully and one rather agreed). All attendees felt that the workshop enhanced their ability to recognize typical sonographic features of ILD, with six fully agreeing and four rather agreeing.

### Future perspectives and perceived barriers and facilitators to the adoption of LUS in ILD

**Fig. 2:**
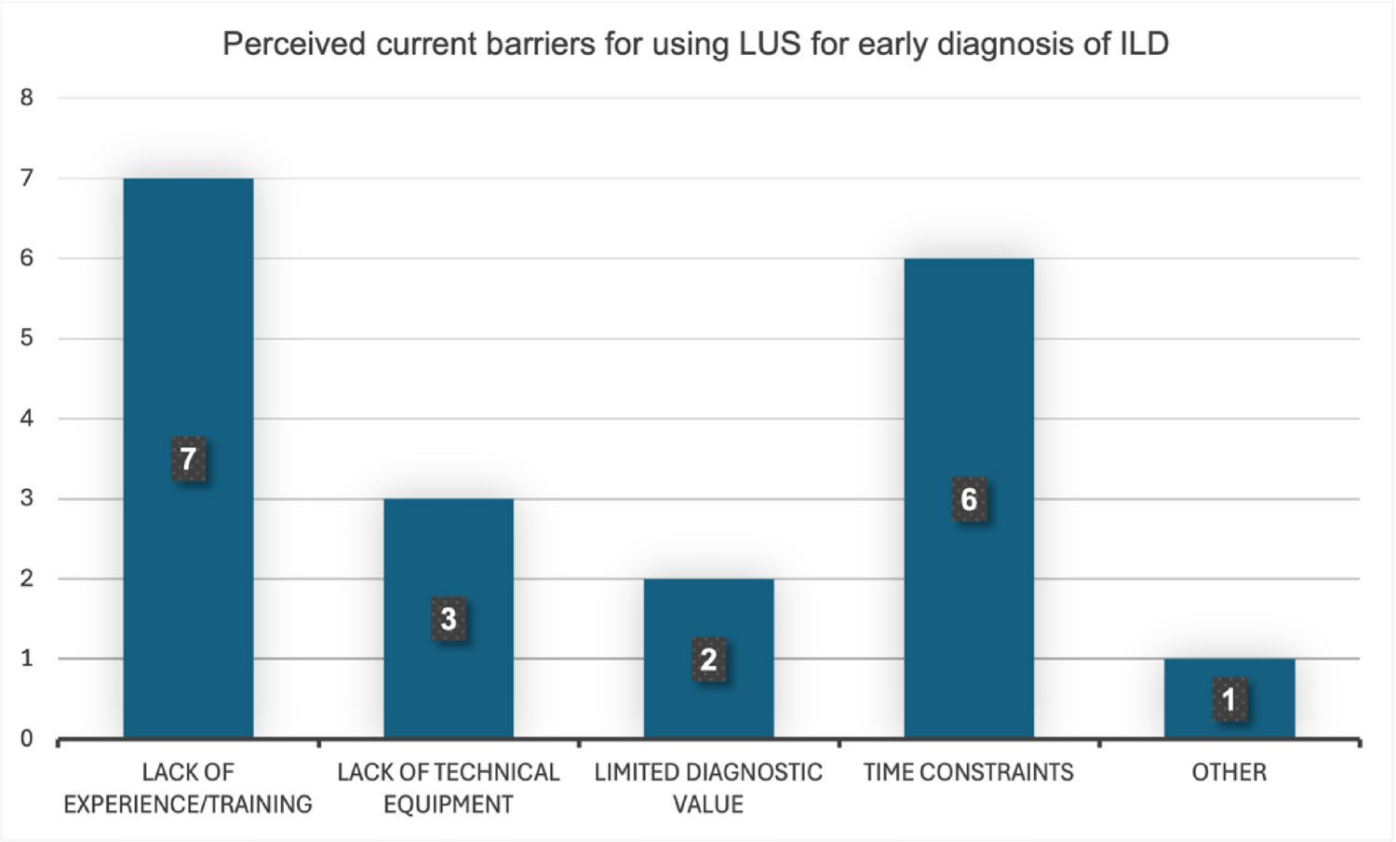
Perceived current barriers to using LUS for early diagnosis of ILD (numbers represent absolute counts of participant responses). Other refers to financial aspects

All participants agreed that workshops like the one conducted help raise awareness of ILD (nine fully agreed, one rather agreed). Six physicians fully and two rather agreed that LUS may play an important role in the early diagnosis of ILD, while two expressed a neutral view. The most frequently reported perceived barriers to usage of LUS in early ILD detection were lack of experience (seven responses) and time constraints (six responses, Fig. 2). Free-text responses regarding potential facilitators for the use of LUS in clinical practice included access to ultrasound equipment (three responses), necessity for standardized protocols and adequate training (two responses, respectively), greater time availability (one response) and existence of reimbursement codes (one response).

## Discussion

Addressing a recommendation highlighted in the current ERS/EULAR CTD-ILD guidelines [5], this study offers novel insights into physician perceptions and perceived clinical relevance of a concise, structured LUS workshop for ILD. To our knowledge, no previous study has evaluated and reported participant perceptions of a combined theoretical and hands-on LUS training program specifically designed for this setting and purposefully involving patients with known ILD.

Consistent with the broader ultrasound education literature, participants in our study perceived the workshop as clinically relevant and applicable to their clinical practice. Ultrasound training programs have repeatedly been associated with improvements in physicians’ knowledge, practical performance and confidence, and are widely perceived as valuable components of medical education [9, 11, 12]. Our findings complement this literature by showing similarly favorable perceptions in a predominantly senior, highly experienced physician cohort.

In this context, the IMPACT-2 study published in 2019 provides an important point of comparison. This study evaluated a structured, four-hour LUS training program exclusively among a very small cohort of eight rheumatologists, with the explicit rationale of enabling ILD screening in rheumatology practice. The educational format was highly comparable to our intervention, combining focused theoretical sessions with hands-on LUS training followed by structured recognition of pathological lung findings. As a notable difference to our study, however, LUS training was performed on healthy subjects. IMPACT-2 demonstrated significant and sustained improvements in ultrasound-related knowledge and practical LUS skills, with gains largely maintained at six months, underscoring the effectiveness of a concise and structured training approach. While the IMPACT-2 investigators reportedly also collected data on perceptions towards prior US experience, training, clinical utility, and attitudes toward LUS, these perception-based outcomes were not presented in detail [13]. Our study therefore complements the IMPACT-2 findings by exploring physician perceptions, perceived educational value, and perceived clinical applicability of LUS training involving patients with ILD. In addition, it examines perceived facilitators and barriers to the clinical use of LUS in a broader and more heterogeneous physician population, including both rheumatologists and pulmonologists.

A key finding of our study is that lack of experience and limited time were perceived as the main barriers to the use of LUS for early ILD detection. This is not surprising given the well-documented time constraints in contemporary clinical practice, where physician-patient encounters are often brief and a substantial proportion of working time is spent on documentation and administrative tasks [14, 15]. 

In this context, LUS may represent a potentially efficient point-of-care tool that can be integrated into routine workflows without substantial additional time burden. Time-efficient scanning protocols may play an additional role. Gutierrez et al. demonstrated that a simplified LUS protocol examining 14 lung intercostal spaces (LIS) correlates well with a comprehensive 50-LIS protocol and with HRCT findings of ILD, while requiring substantially less examination time (8.6 vs. 23.3 min) [16]. This time interval aligns closely with the 5–9 min most of our study participants felt as having realistically available for such an additional examination. These findings further support the clinical practicality of simplified LUS approaches in routine practice.

Importantly, our workshop participants also highlighted the need for standardized scanning protocols and adequate training as key facilitators for adopting LUS into routine clinical practice. This perception closely mirrors the current ERS/EULAR recommendation given for CTD-ILD, which emphasize the need for standardized and prospectively validated LUS screening protocols in asymptomatic at-risk populations [5]. 

Finally, evidence from a recent intensive care unit-based study demonstrates that even a brief, two-hour LUS course combining focused didactic teaching with hands-on training can result in sustained competence over time [10], suggesting that effective and durable learning does not necessarily require extensive training programs. Together with the positive physician perceptions and self-reported educational benefit observed in our study, these findings suggest that brief, structured, and targeted educational formats may represent a practical approach for introducing clinicians to LUS in ILD care.

### Limitations and strengths

This study has several limitations. First, the workshop cohort was very small, limiting statistical validity and generalizability of the findings. In addition, participants predominantly consisted of experienced, hospital-based physicians, which may further restrict applicability to other professional groups or clinical settings. The study also relied exclusively on self-reported outcomes, including perceived knowledge gain, confidence, and intended future use of lung ultrasound (LUS), thereby introducing the potential for response and social desirability bias. Furthermore, no objective assessment of competence or skill acquisition, such as pre-/post-testing or image interpretation analysis, was performed. The cross-sectional post-workshop design additionally precludes conclusions regarding long-term retention of knowledge, sustainability of attitudes, or actual implementation into clinical practice.

Nevertheless, the study also has notable strengths. The small-group workshop format enabled intensive hands-on training with close supervision, which represents a commonly valued educational approach in ultrasound training. Participants originated from different subspecialties and practice settings, allowing diverse perspectives on the role of LUS in ILD. Importantly, the hands-on component was conducted on real patients with different ILD patterns, providing exposure to authentic pathological findings and enhancing the practical relevance of the educational experience. Finally, the study addresses an emerging and clinically relevant educational field and provides exploratory insight into physician perceptions and perceived applicability of LUS in ILD.

## Conclusion

Addressing current recommendations to establish educational and training programs in LUS for ILD, this exploratory study shows positive physician perceptions and perceived educational value of a concise, structured LUS workshop involving actual patients in a predominantly senior physician cohort with regular exposure to ILD. Workshop participants identified lack of experience and time constraints as key perceived barriers, whereas standardized protocols and focused training were viewed as important facilitators for broader clinical adoption of LUS. Overall, these findings suggest that concise and time-efficient educational formats are perceived as valuable by clinicians and could represent a practical approach to increasing familiarity with LUS in ILD care.

## Supplementary Information

Below is the link to the electronic supplementary material.

Supplementary Material 1

## Declarations

### Artificial intelligence (AI)

AI tools were used exclusively for language editing. All AI-assisted content was carefully reviewed, revised where necessary, and verified by the authors.
