# Supplementary material for: Positive physician perceptions of structured lung ultrasound (LUS) training in interstitial lung disease (ILD): a post-workshop survey study
Source: Rheumatol Int. 2026 Jun 3;46(6):127. doi: 10.1007/s00296-026-06191-4 (PMC13233889; doi:10.1007/s00296-026-06191-4)
Supplement: Supplementary file 1 — Supplementary Material 1 [file 296_2026_6191_MOESM1_ESM.docx]

**Positive physician perceptions of structured lung ultrasound (LUS) training in interstitial lung disease (ILD): a post-workshop survey study**

Supplement 1: Post-workshop questionnaire in English

1. What is your age group (single choice)?

- < 30 years
- 30 to 44 years
- 45 to 59 years
- > 60 years

1. How do you identify yourself (single choice)?

- female
- male
- diverse
- prefer not to say

1. What is your medical specialty (single choice)?

- Internal Medicine
- Internal Medicine and Rheumatology
- Pulmonology
- Internal Medicine and Pulmonology
- Other
  1. What is your work environment like (single choice)?
- hospital-based
- outpatient-based
- both
  1. How many years of clinical experience do you have (single choice)?
- < 5 years
- 5 to 9 years
- 10 to 14 years
- 15 to 19 years
- > 20 years
  1. How often do you care for patients with diagnosed interstitial lung disease or patients at risk of developing ILD (single choice)?
- Regularly
- Occasionally
- Rarely
- Never
  1. How often do you use ultrasound as a diagnostic modality in your clinical practice (single choice)?
- Regularly
- Occasionally
- Rarely
- Never
  1. How often do you use lung ultrasound in your clinical practice (single choice)?
- Regularly
- Occasionally
- Rarely
- Never
  1. How much additional time could you realistically allocate to an examination such as lung ultrasound in your current clinical practice (single choice)?
- < 5 minutes
- 5 to 9 minutes
- 10 to 14 minutes
- > 15 minutes
  1. The workshop today was relevant to my clinical practice (single choice).
- Fully agree
- Rather agree
- Neutral
- Rather disagree
- Fully disagree
  1. The content of today’s workshop was practical and easy to understand (single choice).
- Fully agree
- Rather agree
- Neutral
- Rather disagree
- Fully disagree
  1. Today’s workshop significantly improved my knowledge of lung ultrasound (single choice).
- Fully agree
- Rather agree
- Neutral
- Rather disagree
- Fully disagree
  1. After today’s workshop, I feel competent to identify typical sonographic signs (e.g., B-lines, pathological pleural lines) of interstitial lung disease (single choice).
- Fully agree
- Rather agree
- Neutral
- Rather disagree
- Fully disagree
  1. I intend to apply what I learned during today’s workshop in my clinical practice (single choice).
- Fully agree
- Rather agree
- Neutral
- Rather disagree
- Fully disagree
  1. Please rate the following statement (single choice): “I can imagine that lung ultrasound may play an important role in the early diagnosis of interstitial lung diseases in the future.”
- Fully agree
- Rather agree
- Neutral
- Rather disagree
- Fully disagree
  1. Workshops such as this one increase awareness of interstitial lung diseases (single choice).
- Fully agree
- Rather agree
- Neutral
- Rather disagree
- Fully disagree
  1. In your opinion, what are the current barriers to using lung ultrasound in the early diagnosis of interstitial lung diseases (multiple select)?
- lack of experience/training
- lack of technical equipment
- limited diagnostic value
- time constraints
- Other
  1. What factors would facilitate the use of lung ultrasound in your clinical practice (open-ended)?
